# Cellular and molecular interactions underlying the peripheral immune responses to blood-stage malaria infections

**DOI:** 10.1093/immhor/vlag016

**Published:** 2026-04-23

**Authors:** Kieran Tebben, Rosita Asawa, David Serre, Kirsten E Lyke

**Affiliations:** Institute for Genome Sciences, University of Maryland School of Medicine, Baltimore, MD, United States; Department of Microbiology and Immunology, University of Maryland School of Medicine, Baltimore, MD, United States; Institute for Genome Sciences, University of Maryland School of Medicine, Baltimore, MD, United States; Department of Microbiology and Immunology, University of Maryland School of Medicine, Baltimore, MD, United States; Institute for Genome Sciences, University of Maryland School of Medicine, Baltimore, MD, United States; Malaria Research Program, Center for Vaccine Development and Global Health, University of Maryland School of Medicine, Baltimore, MD, United States

**Keywords:** host-pathogen interactions, immunology, malaria

## Abstract

Malaria, caused by *Plasmodium* species, is one of the most widespread illnesses globally, affecting millions of individuals each year. The complex life cycle of these parasites requires a multifaceted approach from the human immune system to respond to infection. Additionally, *Plasmodium* parasites have coevolved in primates and developed numerous immune evasion mechanisms to escape human immune defenses. Here, we provide an up-to-date review of the human immune responses to blood-stage malaria, as well as the parasite immune evasion mechanisms during this part of the life cycle.

## Malaria

Malaria is a vector-borne disease caused by infection with 1 of 5 species of protozoan *Plasmodium* parasites following the bite of an infected female *Anopheles* mosquito.^1^ Approximately 3 billion people, almost half of the world’s population, are at risk for malaria,^2^ making this disease one of the leading public health issues worldwide. In 2023, there were 263 million global cases of malaria, with the most severe cases and the highest burden of disease occurring in sub-Saharan Africa and caused by *Plasmodium falciparum.*^2^

Malaria is characterized by cyclical fevers,^3^ that recur every 24 to 36 h depending on the parasite species,^1^ and flu-like symptoms caused by the asexual replication of the parasites within red blood cells. *P. falciparum*, the dominant species in sub-Saharan Africa, can also lead to severe symptoms, particularly in children under 5 yr of age,^1^^,^^4^ which can be classified into 3 main syndromes: severe malarial anemia, cerebral malaria, and acute respiratory distress.^5^ If not treated rapidly, severe malaria is often deadly^4^^,^^5^ and is a leading cause of mortality in children under 5 yr of age in sub-Saharan Africa.^5^

All human-infecting *Plasmodium* species share the same life cycle, including development in a mosquito vector and human host.^6^ Within the mosquito, *Plasmodium* parasites develop from male and female gametes in the midgut to transmissible salivary gland sporozoites within 2 wk.^1^ When an infective mosquito takes a blood meal, sporozoites are injected into the skin of the mammalian host and rapidly travel through the blood stream to the liver.^1^ After infection of a hepatocyte, sporozoites develop into liver schizonts, which eventually rupture and release merozoites in the circulation.^1^ Merozoites infect red blood cells (RBCs) and begin the intraerythrocytic development cycle (IDC), first developing into ring stage parasites (rings).^1^ Within the infected red blood cells (iRBCs), the IDC continues as rings develop into trophozoites and then blood schizonts.^1^ Schizonts eventually rupture and release merozoites into the circulation to infect new RBCs and begin a new IDC.^1^ This IDC leads to an increase in parasitemia up to 32× each cycle or every 24 to 72 h (depending on the *Plasmodium* species).^7^ A subset of blood-stage parasites become sexually committed and develop into male or female gametocytes^8^ that, when ingested during a blood meal, lead to transmission to the mosquito.

Immunopathology induced by the asexual blood-stage parasites is responsible for all symptoms of malaria.^1^^,^^5^^,^^9^ The immunopathology of symptomatic malaria is complex and multifactorial but involves endotoxins released into the circulation when schizonts rupture iRBCs and TNFα secreted by macrophages upon ingestions of merozoites and iRBCs.^5^ Symptoms of malaria typically arise when inflammation reaches a critical, although undefined, threshold and the patient feels ill.^5^

## Immune responses to the *P. falciparum* IDC

**Figure 1:**
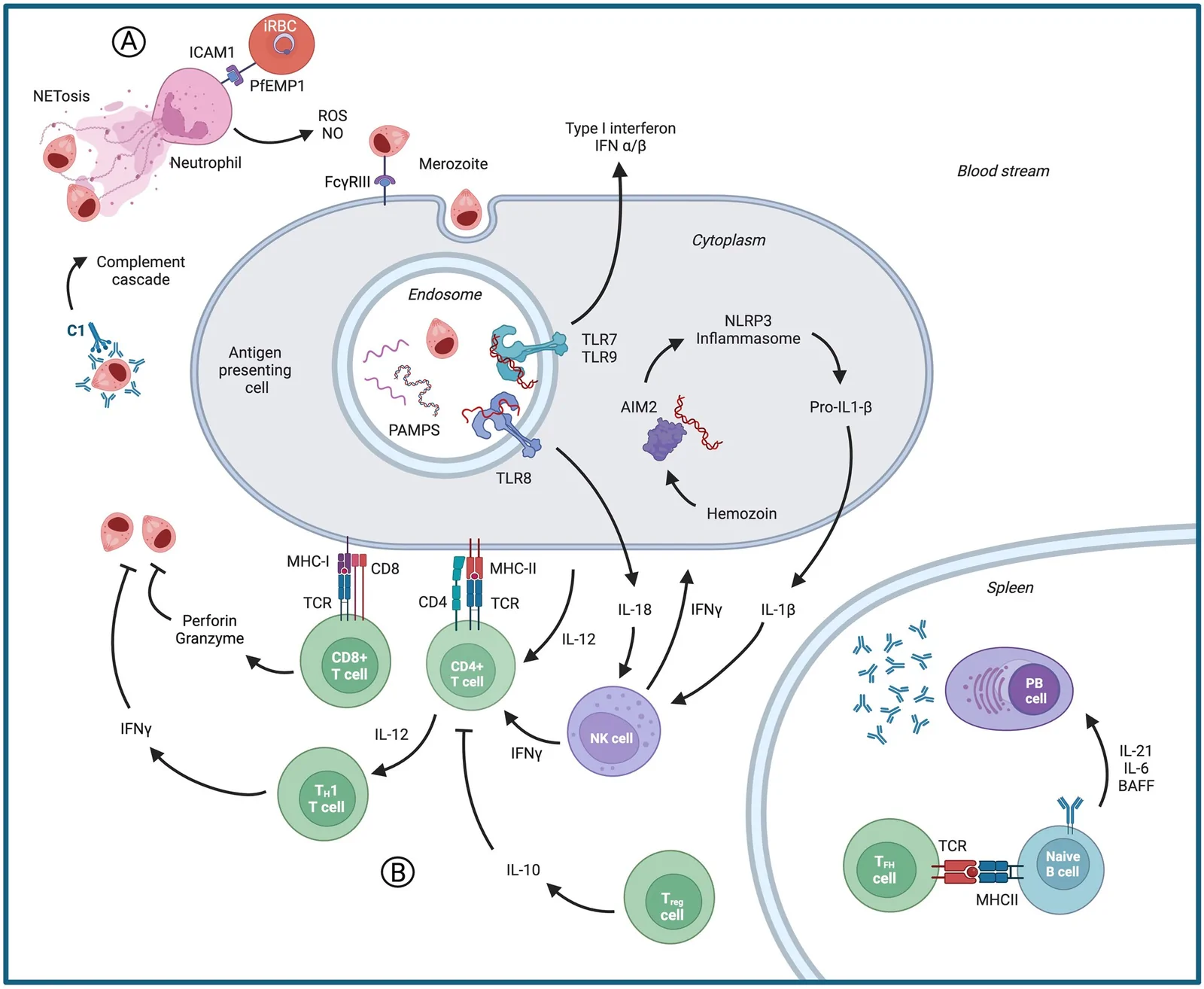
Innate and adaptive immune response to blood-stage *P. falciparum*. (A) Innate immune response: iRBCs are recognized by neutrophils through binding of intercellular adhesion molecule 1 (ICAM1) to PfEMP1s expressed on the surface of iRBCs leading to release of reactive oxygen species (ROS)/nitric oxides (NOs). Activated neutrophils degranulate to release NETs to kill extracellular merozoites. Circulating antibodies bind to merozoites and recruit C1 to activated the complement pathway. Extracellular merozoites are phagocytosed by antigen-presenting cells (APCs) via FCγRIII binding leading to release of pathogen-associated molecular patterns (PAMPs) in the endosome. TLR7, TLR8, and TLR9 recognize PAMPs to stimulate a type I IFN response and release of IL-18. AIM2 in the cytosol, enhanced by hemozoin, recognizes double-stranded DNA and leads to the generation of IL-1β, which activates NK cells to release IFNγ. (B) Adaptive immune response: APCs activate naïve CD4+ T cells through MHC class II (MHC-II)–TCR interactions stabilized by CD4. The secondary release of IL-12 from APCs polarizes CD4+ T cells toward a Th1 phenotype, which release IFNγ to directly reduce parasitemia. CD8+ T cells are activated through MHC-I–TCR binding, stabilized by CD8, to release perforin and granzyme, which directly target extracellular merozoites. In the spleen, Tfh cells stimulate naïve B cells through MHC-II–TCR interactions resulting in differentiation to antibody-producing plasma cells.

**Figure 2:**
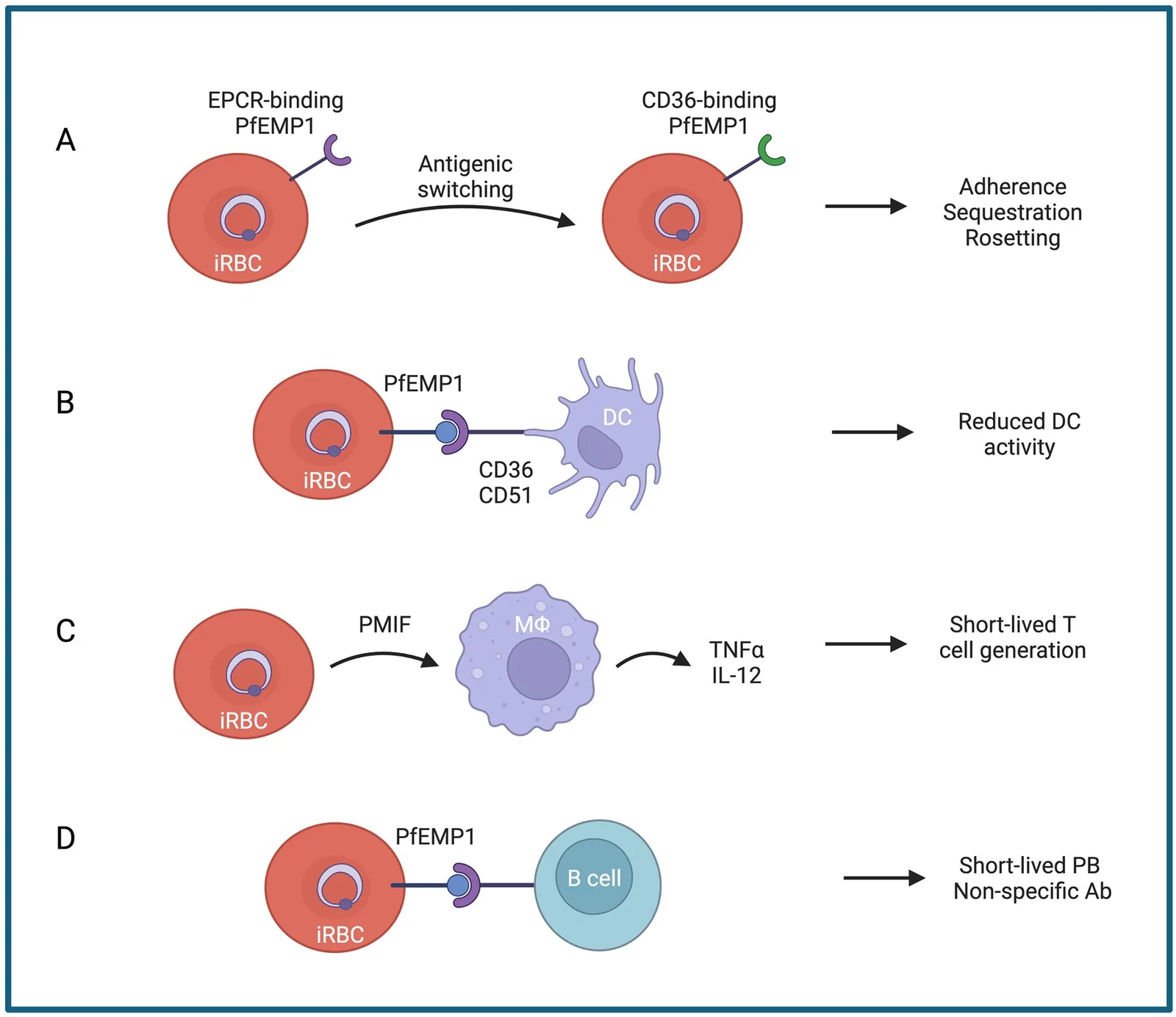
Examples of blood-stage immune evasion mechanisms in *P. falciparum*. PfEMP1s are a large VSA family within *P. falciparum* that boast several mechanisms for immune evasion. (A) Host immune pressure triggers PfEMP1s on the surface of iRBCs to switch between antigenically distinct PfEMP1s, referred to as antigenic switching, leading to continued PfEMP-mediated iRBC adherence, sequestration, and rosetting. (B) PfEMP1 on the surface of iRBCs bind to CD36/CD51 on DCs leading to reduced DC activity. (C) iRBCs secrete peripheral blood mononuclear cell–derived factor (*Plasmodium* macrophage migration inhibitory factor), which stimulates macrophages to produce TNFα and IL-12, skewing the immune response to develop short-lived T cells. (D) iRBCs interact directly with B cells through PfEMP1 to skew the humoral response toward short-lived plasmablast (PB) cells and nonspecific antibody (Ab) generation. EPCR, endothelial protein C receptor.

The complex life cycle of *Plasmodium* parasites within the human host leads to unique immune responses to each developmental stage.^10^^,^^11^ In this review, we focus on the asexual blood stages (i.e. the IDC) of *P. falciparum* that induce robust, systemic inflammation and are responsible for all malaria symptoms.^1^^,^^10^ The immune response to the liver stages, which were long considered asymptomatic, were recently reviewed by Abuga et al.^12^ Blood-stage parasites interact with a variety of immune cells from the innate and adaptive immune systems, described in more detail subsequently. Some immune memory also develops to these stages over time and repeated exposures, leading to a reduction in symptom severity in older patients, but rarely providing sterile immunity.^10^^,^^13–15^ Here, we briefly review the major cell types of the innate (Fig. 1A) and adaptive (Fig. 1B) immune system that are responsible for the anti-*Plasmodium* immune response, as well as mechanisms by which *P. falciparum* evades this immune detection (Fig. 2).

## Innate immune responses

### Complement

The complement system is an ancient component of the innate immune system, predating jawed vertebrates,^16^ and one of the first arms of the immune response to encounter parasites in the blood.^17^ Merozoites, the only extracellular stage within the IDC, can fix complement directly on the cell surface and are susceptible to complement-mediated lysis and killing, particularly after complement fixation via the classical pathway, with circulating antibodies interacting with merozoite surface antigens such as merozoite surface proteins (MSPs),^18^ PfRH5, and AMA1.^19^^,^^20^ Complement fixation on the merozoite surface can also enhance phagocytosis by phagocytes and antigen-presenting cells^21^ and recruitment of additional innate immune cells.^17^ In the absence of other complement components, fixation of C1q/antibody complexes to the merozoite surface can also efficiently prevent merozoite invasion of RBCs.^19^

The more mature, intracellular, stages of the *P. falciparum* IDC remodel the surface of the iRBC with parasite proteins that can be recognized by circulating antibodies^22^ but overall are less susceptible to complement-mediated killing. Despite the presence of antigenic targets, prior work has demonstrated relative protection of iRBCs from complement activation, and particularly complement-mediated lysis.^23^ One of the most well-characterized and prevalent targets of anti-*P. falciparum* antibodies on the iRBC surface is *P. falciparum* erythrocyte membrane protein 1 (PfEMP1), but extreme sequence variation in PfEMP1 between different parasites challenges the development of specific antibodies.^24^ Additionally, despite evidence of opsonization,^25^ the structure of PfEMP1s on the RBC surface^25^ and binding by IgM^26^^,^^27^ seems to preclude complement activation on iRBCs.

### *Innate immune sensing of* P. falciparum

Innate immune sensing of *Plasmodium* parasites, including *P. falciparum*, has been extensively reviewed elsewhere.^28^ Briefly, some of the most well-characterized *P. falciparum* molecules recognized by the host cells (pathogen-associated molecular patterns) are glycosylphosphatidylinositols.^29^^,^^30^  *Plasmodium* glycosylphosphatidylinositols are recognized by Toll-like receptor 2 (TLR2)^30^ and lead to the production of proinflammatory cytokines.^29^ Upon parasite digestion by phagocytes, *P. falciparum* DNA is sensed by TLR7 and TLR9^31^ on the inner membrane of endolysosomes or by the cytosolic DNA sensor, AIM2.^32^ Signaling through TLR7 and TLR9 initiates a type I IFN response, leading to the release of proinflammatory cytokines IFNα and IFNβ.^33^ Sensing through AIM2 is enhanced by hemozoin, a crystalline byproduct of hemoglobin digestion by the parasites, which can itself be sensed by the NLRP3 inflammasome,^32^ both of which lead to cleavage and release of IL-1β.^34^ Similarly, parasite RNA can be sensed by TLR8 on the inner membranes of monocyte phagolysosomes^35^^,^^36^ and leads to the release of IL-1β and IL-18, leading to the downstream release of IFNγ by natural killer (NK) cells.^35^ Together, this proinflammatory cytokine environment contributes to triggering fever and the flu-like symptoms of malaria.

### Neutrophils

Neutrophils, the most abundant white blood cell, rapidly respond to blood-stage *Plasmodium* parasites.^37–39^ Neutrophils can kill free merozoites or iRBCs via phagocytosis, or by releasing toxic molecules such as reactive oxygen species or neutrophil extracellular traps (NETs).^39^ Neutrophils can interact directly with iRBCs via intercellular adhesion molecule 1 on the neutrophil surface binding to CD36-binding PfEMP1s, leading to phagocytosis and killing of the iRBC.^40^ Phagocytosis of merozoites or iRBCs can be enhanced by complement-mediated opsonization or antibody binding to parasite antigens on the iRBC surface that interact with complement and Fcγ receptors on neutrophils^37^^,^^41^ or by release of proinflammatory cytokines.^42^ Phagocytosis then triggers killing of the parasites or iRBCs by reactive oxygen species^42^ or microbicidal granule proteins^43^ within the neutrophil. Like phagocytosis, parasite-specific antibodies can also enhance reactive oxygen species release and neutrophil-mediated parasite killing (i.e. antibody-dependent respiratory burst).^44^

In addition to phagocytosis, neutrophils can immobilize and kill parasites through degranulation of their genetic material and antimicrobial granules as NETs.^45^^,^^46^ Parasite molecules such as uric acid (a byproduct of purine salvage),^47^ as well as proinflammatory cytokines,^48^ can increase NET production. NETs have been reported to be important for not only control of parasitemia,^49^ but also exacerbation of immunopathology.^50^ While neutrophils are essential for initial control of parasitemia and for reducing the pathogen load, their activation has been linked to immunopathology^38^^,^^39^^,^^51^ and severe malaria,^37^^,^^52^ potentially via inflammation-induced endothelial damage in the tissues, although the precise mechanisms remain unclear.

### Monocytes and macrophages

In the circulation, monocytes are also major contributors of phagocytosis and killing of iRBCs, with 2 subsets of cells, intermediate/inflammatory (CD14+CD16+) and nonclassical (CD16+) monocytes, being particularly efficient.^53–55^ Nonopsonic phagocytosis is mediated by monocyte TLR2 and TLR4 stimulation and interactions of the monocyte surface protein CD36 with PfEMP1s on the iRBC surface.^56^ Opsonic phagocytosis requires opsonization of merozoites or iRBCs with antibodies and complement proteins, which interact with FcγRIII on the monocyte surface, triggering phagocytosis.^57^ While both mechanisms of phagocytosis can lead to parasite killing, only opsonic phagocytosis leads to downstream monocyte activation and release of proinflammatory cytokines such as TNF and IL-1β.^55^^,^^58^

Because macrophages primarily exist within tissues, it is difficult to characterize their interactions with blood-stage parasites in humans and many studies so far have relied on mouse models to speculate about their function during human infections.^55^ Within tissues, macrophages typically interact with sequestered iRBCs (containing mature asexual stage parasites). Similarly to monocytes, macrophages can perform nonopsonic phagocytosis via CD36^59^ or opsonic phagocytosis via antibodies and complement.^60^ Again, only opsonic phagocytosis is associated with macrophage activation and proinflammatory cytokine production.^60^

Similarly to neutrophils, proinflammatory cytokine release by monocytes and macrophages can contribute to both control of parasite load via further activation of the phagocytes,^61^^,^^62^ as well as immunopathology^55^ and severe malaria syndromes.^52^^,^^63^

### Dendritic cells

Dendritic cells (DCs) are an important bridge between the innate response and both branches of the adaptive immune system in response to blood-stage malaria.^64^ Generally, DCs phagocytose *P. falciparum* merozoites or iRBCs similarly to monocytes and macrophages. After phagocytosis, DCs become activated, increasing expression of the major histocompatibility complex (MHC) class 1 or 2 and costimulatory molecules on their surface.^64^ After activation, DCs travel to the spleen or secondary lymphoid tissues^64^ to initiate T cell responses via antigen presentation, costimulation, and release of proinflammatory cytokines, which initiates the adaptive immune response and/or recruits and activates additional innate immune cells to the site of infection.^64^ There are still little data from human infection, but in mouse models, different subsets DCs have been shown to induce both parasite-specific CD4 and CD8 T cell responses^65^^,^^66^ after interaction in the blood with parasites or iRBCs. While peripheral CD4 T cell responses can lead to control of parasitemia and disease, initiation of a peripheral CD8 T cell response has been associated with cerebral malaria pathogenesis.^65^

DC responses to *P. falciparum* vary with transmission intensity and their efficacy in generating an effective immune response for clearance of the parasites is directly impacted by the parasites themselves. In high-transmission settings, a particular subset of highly MHC class II–expressing DCs (by flow cytometry identification of HLA-DR), BDCA-3^+^ cDC1s,^67–69^ was found to be expanded after infection, while other DC subtypes, showed generally reduced expression of MHC class II,^68^^,^^70^ detected by flow cytometry, and a reduced ability to stimulate T cells.^67^ The functional consequences of this observation remain unclear but warrant further investigation with human cohorts. In low-transmission settings, DCs isolated from peripheral blood of *P. falciparum–*infected patients were noted to be apoptotic and unable to be activated or to stimulate T cell responses.^71^ Controlled human malaria infection studies have also observed a decrease in expression of MHC class II on certain DC subsets, along with reduced IL-12 production,^72^ suggesting reduced ability to productively bridge the innate and adaptive immune systems. Additional work has suggested a critical threshold of parasite biomass to induce this DC dysregulation.^73^ Crosstalk between different DC subsets may also be key to their response to *P. falciparum*,^74^^,^^75^ but further work is necessary to fully disentangle the relationship between protective immune responses generated by DCs during infection and their dysregulation.

### NK cells

NK cells have 3 major pathways of activation: cytokine activation via IL-12 and IL-18, antibody-dependent cell-mediated cytotoxicity (ADCC), and the loss of inhibitory signal by damaged or diseased cells. In malaria infections, a positive association between IFN-γ production and protection from malaria^76–78^ brought attention to the interplay between NK cells and *P. falciparum* infection. NK cells can directly reduce parasitemia by targeting and destroying iRBCs through antibody-dependent and antibody-independent mechanisms. In the presence of IgG specific for *P. falciparum* antigens on the iRBC surface, NK cells can adhere to iRBCs via leukocyte integrin αLβ2 before releasing perforin and granzyme to selectively lyse iRBCs, exposing the parasitophorous vacuole for phagocytosis by monocytes.^79^

These cells can also, indirectly, lead to destruction of iRBCs through the release of IFNγ,^80^ which activates macrophages and leads to increased phagocytosis of iRBCs.^35^ Additionally, expression of PD-1 on NK cells in malaria-exposed individuals is associated with diminished NK cytotoxicity but improved ADCC, indicating that PD-1 may contribute to the skewing of NK cells toward ADCC activity seen during malaria infection.^81^

Recent work has highlighted a phenotypically and transcriptionally unique population of NK cells that do not express CD56, a classical NK cell marker, that appear to be important to parasitemia control.^82^^,^^83^ These cells were found to expand rapidly upon repeated malaria exposure, and their expansion was associated with protection against high parasite density and symptomatic malaria.^83^ Interestingly, maintenance of this population appears to be exposure dependent and rapidly declines without *Plasmodium* exposure.^83^ Further work found that activation of these unique NK cells is influenced by both host and parasite factors, again highlighting the complexity of the interplay between *P. falciparum* and the human immune system.^82^ NK cells isolated from malaria-exposed Ugandan donors displayed enhanced ADCC and in vitro studies measured enhanced NK cell degranulation in response to *P. falciparum* schizonts when cultured with plasma from donors in high-transmission areas.^82^ This reinforces the impact of *P. falciparum* exposure on influencing the NK cell response^82^ and highlights the need for further research to fully characterize both how NK cells contribute to the anti-*Plasmodium* response and how parasite exposure influences this response to better understand how to exploit these interactions for treatment or prevention of malaria.

While traditionally classified as innate immune cells, NK cells have also been shown to demonstrate a “memory” or “memory-like” immunological response leading to increased cytokine production and cytotoxic effects upon restimulation.^84^ This memory-like phenotype, as well as their potential exposure-dependent activation, is similar to how the adaptive immune system interacts with *P. falciparum*.

### *P. falciparum* blood-stage evasion of the innate immune response

Immune evasion mechanisms in *Plasmodium spp.* have been extensively reviewed elsewhere,^11^^,^^85^^,^^86^ but here we highlight the main mechanisms of evasion of the innate immune system in humans. In order to survive in circulation, parasites must avoid the early innate phase of the immune response such as complement activation and, ultimately, opsonization and phagocytosis. During the IDC, the RBC, which does not express MHC molecules, serves as an immunological sanctuary. This is a key mechanism of immune evasion, however the merozoites, the only extracellular blood stage, remain susceptible to immune attack.^87^ Merozoites recruit complement inhibitory factors, C1-INH and factor H,^88^ to their surface to avoid complement fixation and activation. They also avoid detection by circulating antibodies, which can block invasion and/or lead to complement activation, through sequence variation of antigens on their surface and through functional redundancy of proteins to preserve invasion machinery if one molecule is blocked.^87^

During the intraerythrocytic stages of the IDC, *P. falciparum* iRBCs also recruit complement inhibitory factors, factor H,^89^ and plasminogen^90^ to avoid complement fixation. Antibodies against PfEMP1, the major surface antigen of *P. falciparum* iRBCs, seem to be unable to fix and activate complement,^25^ potentially due to the structure of knobs on which PfEMP1 is presented.^25^ Infected RBCs also form rosettes, or clusters of uninfected RBCs surrounding iRBCs, via binding of iRBCs to complement receptor 1 on the uninfected RBC surface,^91^ which physically hide parasite surface antigens from circulating antibodies or immune cells (Fig. 2A).^92^ While avoiding complement activation, iRBCs must also avoid phagocytosis by circulating phagocytes or resident phagocytes in the spleen. By avoiding complement activation, parasites reduce opsonization, which prevents their efficient phagocytosis in circulation. *P. falciparum* parasites, uniquely, sequester in the tissues later in the IDC via interactions of PfEMP1 with endothelial surface markers on the tissue microvasculature,^92^^,^^93^ which limits their interaction with phagocytes to tissue-resident cells. Tissue sequestration also prevents iRBCs from being filtered through the large population of resident phagocytes in the spleen.^92^

In addition to simply avoiding immune activation, *Plasmodium* parasites also actively dampen the innate immune response. *P. falciparum* iRBCs express high levels of CD47 on their surface, which interacts with inhibitory receptors on the macrophage surface (e.g. SIRPα) and decreases phagocytosis.^94^ Additionally, during infection, complement receptor 1 expression is reduced on monocytes and macrophages by an unknown mechanism, reducing complement-mediated phagocytosis.^91^ Monocyte phagocytosis of iRBCs is inhibited by the production of insulin growth factor binding protein 7.^95^ Even after successful phagocytosis, phagocytes and neutrophils have a limited capacity to digest and remove iRBCs, due to hemozoin toxicity, before becoming overloaded with this pigment and unable to perform their function in clearing parasites.^96^^,^^97^

Dysregulation of the DC response is a critical mechanism of *P. falciparum* immune evasion, as the initiation of the adaptive immune response critically depends on an appropriate initial DC response to the parasites (Fig. 2B). *P. falciparum*’s ability to dysregulate DCs is not yet completely understood, but several mechanisms have been proposed. Hemozoin exposure from iRBCs can partially impair DC maturation.^98^ Direct interaction of iRBCs and DCs also seems to impair DC maturation, antigen processing, and T cell stimulation,^99^^,^^100^ but the mechanism underlying this impairment is still unknown. The mechanisms by which *P. falciparum* induce DC apoptosis in vivo, described previously, are not yet understood but will be key to understanding protective antimalarial immunity and the improvement of existing vaccines.

## Adaptive immune responses to blood-stage *Plasmodium* parasites

### CD4+ T helper cells

Peripherally circulating CD4+ T helper (Th) cells play a central role in responding to blood-stage *P. falciparum* (while liver-resident CD8+ T cells are critical for the pre-erythrocytic immune responses in the liver).^101^ Innate immune cell detection of different parasite antigens by different receptors (reviewed previously) leads to particular cytokine production according to the specific antigen and receptor bound?. Cytokines released from antigen-presenting cells polarize CD4+ Th cells toward various phenotypes, which could be skewed by the cell type and mechanism by which these cells are being activated. These phenotypes have been reviewed in greater detail elsewhere^102^^,^^103^; we briefly review the major phenotypes subsequently. *P. falciparum–*specific antigen-presenting DCs release cytokines such as IL-12 and IL-6 (predominantly via TLR2 and TLR8 sensing of iRBCs),^104^ polarizing Th cells toward a Th1 phenotype.^103^ Th1 polarized cells play a large role in T cell–mediated control of blood-stage *P. falciparum*, primarily through release of IFNγ^74,.105,106^ While the exact mechanism by which IFNγ release contributes to parasitemia control is not yet clear,^103^ data from mouse models suggest that this cytokine activates macrophages, increasing their ability to phagocytose iRBCs.^107–109^ A subset of these cells also produce macrophage colony-stimulating factor, further promoting the activity of macrophages in controlling parasitemia.^110^ While IFNγ seems to be critical in reducing parasite burden, it can also contribute to pathogenesis by impairing parasite-specific B cell responses.^103^ A unique subset of Th1 cells produce both proinflammatory IFNγ and anti-inflammatory IL-10 in response to stimulation from IL-27^111^ and type I IFNs.^112^ While their precise role in the antimalarial immune response remains elusive, a higher abundance of these cells has been associated with increased risk of malaria in pregnancy,^113^ and IL-10 production from these cells has been associated with both reduced immunopathology^114^^,^^115^ and impaired ability to control parasitemia.^116^

While Th1 is the dominant phenotype in response to *P. falciparum* blood stage, the role of other Th phenotypes is not completely understood. Th2 polarized cells release IL-4, which can support B cell class switching,^117^^,^^118^ but their role in *P. falciparum* blood-stage infection is yet to be confirmed. Th17 polarized cells capable of production of IL-17, which recruits neutrophils,^119^ have been identified in 1 Malian cohort with *P. falciparum,*^120^ but their role in the antimalarial immune response is still unclear.

### T follicular helper cells

T follicular helper cells (Tfh) bridge the cellular and humoral arms of the adaptive immune system and can be identified by expression of BCL-6, CXCR5, and PD1.^121^ After their own activation and development, promoted by IL-6 and IL-21, Tfh cells interact with B cells via production of IL-21^122^ and expression of ICOS^123^^,^^124^ to stimulate B cell maturation and survival within the germinal center of the secondary lymphoid tissues.^125^ During malaria, Tfh cells have been identified as critical in promoting the B cell–mediated generation of *P. falciparum* antigen-specific antibodies, which are important for controlling parasitemia,^126^^,^^127^ and for the differentiation of naïve B cells into memory B cells (MBCs) and long-lived plasma cells,^103^^,^^127^ which are critical for long-lasting immunity to the parasites.

In addition to supporting B cell responses, *Plasmodium-*specific Tfh cells can adopt partial phenotypes from other T cell subsets.^128^ Tfh cells with a Th1-like phenotype (Tfh-Th1) secrete IFNγ,^128^ a cytokine linked to parasitemia reduction and promotion of protection upon reinfection,^129^ but, surprisingly, Tfh-Th1cells have been shown to provide compromised or “atypical” B cell help during infection.^128^ Both IFNγ^112^^,^^124^^,^^130^ and type I IFNs^124^ have somewhat paradoxically been linked to impaired Tfh function in mouse models. Controlled human malaria blood-stage infections have demonstrated increased expression of CXCR3 on Tfh-Th1 cells and an association with ineffective antibody formation and reduced malaria immunogenicity.^131^ Tfh cells with a regulatory T cell phenotype can produce IL-10 in the germinal center.^132^ Interestingly, recent work has shown that extrafollicular IL-10 is critical for the Tfh development and maturation,^133^ but the role of this cytokine within the germinal center during malaria is still unclear and could contribute to the poor memory response generated by these parasites.

Most of our current knowledge of germinal center Tfh cells comes from mice due to sampling accessibility. However, human studies have demonstrated activation of Tfh cells toward a Th1 phenotype in the circulation after *P. falciparum* exposure,^128^ and activation of this subset increases with age.^134^ These cells seem to be poorly able to activate naïve B cells and produce IFNγ,^128^ which has been linked to development of atypical, exhausted MBCs^135^ and short-lived plasma cells.^136^ Some studies have linked circulating Tfh cells with a Th2 phenotype to functional antibody production,^127^^,^^131^ but further work is needed to understand how this phenotype is induced. Interestingly, only Th17-Tfh cells have been associated with subsequent protection from malaria^134^ in humans, although their precise function during malaria is not yet characterized.

### Regulatory T cells

Regulatory T cells (Tregs) act to regulate the inflammation induced by other cell types in response to infections by releasing anti-inflammatory cytokines such as IL-10, and limit immunopathology that can be caused by chronic inflammation.^137^ Their role in malaria is still controversial and seems to antagonize the productive immune control of the parasites.^138^ In humans, the Treg population expands during the *P. falciparum* blood stage, and larger numbers of Tregs are associated with worse clinical outcomes.^139^ Some speculate that expansion of Tregs during severe infection is a consequence of the increased parasitic load, which results in paradoxical immune suppression and worse infection control.^103^ Some research suggests a beneficial role for Tregs_,_ mitigating some of the sequelae of malaria. People of Peuhl ethnicity in West Africa have been shown to have a genetic enhancement in Treg production, correlating with enhanced protection against malaria compared with other ethnicities.^140^^,^^141^ Recent work has also shown that dihydroartemisinin, a common antimalarial drug, can also induce the expansion of these cells.^142^ Future work is important to understand how and why this population expands during malaria and their functional consequences, particularly as it might relate to age of the infected individual.

After expansion, Tregs secrete IL-10, which leads to decreased production of proinflammatory cytokines from other cell types^139^ and can limit symptoms caused by systemic inflammation, but also allows parasite growth to continue. The precise mechanisms of Treg function during malaria are still incompletely characterized, but the expression of CTLA4 seems to impair germinal center reactions,^143^ and further work is needed to fully understand the role of these cells in malaria.

### γδ *T cells*

γδ T cells are a specialized subset of unconventional T cells that perform both innate-like and adaptive-like functions, including the production of proinflammatory cytokines, MHC-independent antigen presentation, cytotoxic killing, and promotion of DC and B cell maturation.^144^^,^^145^ Their role in malaria immunity is a rapidly expanding field of research and much remains unknown, but we have briefly summarized some main findings here. These T cell receptor (TCR)–containing cells were originally considered to be innate-like with no ability to form long-term responses, but long-term elevation in the proportion of peripheral γδ T cells has been found in *Plasmodium*-exposed humans following both irradiated sporozoite vaccination^146^ or controlled experimental infection of human volunteers.^147^ These cells are characterized by the specific γ and δ TCR chains they express and particular subsets rapidly proliferate following a primary *P. falciparum* infection to produce a robust proinflammatory cytokine response^148^^,^^149^ and granulysin-containing cytotoxic granules,^150^^,^^151^ associated with protection from clinical malaria.^152^^,^^153^ However, the exact mechanism behind γδ T cell expansion, including which subsets expand and which do not, and how this leads to protection from malaria is still poorly understood. Initial exposure is characterized by a dominant innate-like vδ2 T cells. Repeated exposure to *Plasmodium* parasite leads to expansion of vδ1 T cells, characterized by waves of clonal selection in the vδ1 TCR repertoire and differentiation into an adaptive vδ1effector morphologies. These cells can both recognize parasite phosphoantigens and trigger an adaptive response, and also directly lead to parasite killing via phagocytosis of iRBCs and production of cytotoxic granules. However, diminishing expansion of these cells and loss of functional activity over time has also been observed, representing a potential immunological tolerance mechanism contributing to clinical manifestations of malaria.^152^^,^^154^ Further work is currently being done and is much needed to fully understand how these cells contribute to immune response to malaria and how the parasites interact with them in the peripheral blood.

### B cells

B cells are responsible for the humoral response to *Plasmodium*, which primarily involves the generation and secretion of *Plasmodium* antigen-specific antibodies.^155^ Naïve B cells interact with *Plasmodium* antigens in the peripheral blood or the immune follicles via the B cell receptor and become activated.^155^ After activation, B cells travel to the secondary lymphoid tissues where they undergo clonal expansion and further mature.^156^ In the secondary lymphoid tissues, DCs and Tfh cells support B cell maturation toward two lineages, plasmablasts or germinal center B cells (GCBs),^14^ by providing important cytokines (such as IL-21, IL-6, and BAFF) and costimulatory molecules (such as CD40L and ICOS).^155^

B cells that mature into plasmablasts rapidly produce and secrete antibodies within days of encountering a pathogen but primarily produce low-affinity IgM type antibodies.^14^ These antibodies can help to control pathogens while more specific B cell responses develop over weeks to months but are short-lived and are not found in the blood after the infection has cleared.^14^ In mouse models of malaria, plasmablasts expand during infection but seem to contradict the development of a productive immune response.^136^ These cells have been shown to act as a nutrient sink, impairing the development of a productive humoral immune response.^136^

GCBs enter a specialized area within the germinal center and can further mature into MBCs or long-lived plasma cells to provide lasting humoral immunity to *Plasmodium* infection.^14^ In the germinal center, GCBs form sustained interactions with Tfh cells to facilitate their development to either cell type,^14^ and undergo somatic hypermutation and class-switch recombination to improve the affinity of their secreted antibodies to their antigen.^155^ Additionally, GCBs primarily express higher affinity IgGs rather than IgM.^137^ GCBs that differentiate to long-lived plasma cells reside in the bone marrow where they stably secrete anti-*Plasmodium* antibodies for years after infection.^14^ MBCs remain in the peripheral blood or secondary lymphoid tissues for years after infection and can rapidly expand and activate upon secondary encounters with their antigen (discussed more subsequently).^14^ Several mechanisms have been proposed to explain the inefficient generation of a humoral immune response to *P. falciparum*, including poor Tfh help and aberrant dampening of the immune response by Th1 skewed Tfh cells and Tregs,^14^ but work is still needed to completely understand these mechanisms and their consequences. Aside from inefficient development in the germinal center, malaria has also been shown to dysregulate the B cell niche in the bone marrow by reducing CXCL12 and IL-7 production,^157^ which impairs the survival and subsequent development of B cell progenitors and plasma cells.

### Antibodies

Antibodies are known to play a critical role in combating blood-stage *P. falciparum* infection and controlling the development of clinical symptoms. The peripheral immune response to *P. falciparum* is driven mainly by the IgG antibody response, in particular by the IgG1 and IgG3 subclasses that have high-affinity Fc receptors.^158^ Antimalaria antibodies act in several ways to control infection including the neutralization of *Plasmodium* ligands,^159^ induction of complement,^18^ and enhanced phagocytosis of merozoites.^160^ Protection from malaria has been strongly associated with high antibody levels to several blood-stage and pre-erythrocytic stage proteins, the most promising of which includes AMA1. Antibody titers specific for AMA1, generated from any cell type, have been associated with clinical protection from malaria symptoms,^161^^,^^162^ and anti-AMA1 antibodies have been shown to inhibit invasion of RBCs by merozoites.^163^

## Development of adaptive immune memory to blood-stage parasites

Both the cellular and humoral branches of the adaptive immune response have the capacity to develop into pathogen-specific memory T or B cells that remain in circulation after an infection has resolved and can respond and expand rapidly upon secondary exposure, in some cases providing sterile protection from subsequent infection with a pathogen.^137^ Memory responses to malaria were first hypothesized because of the development of clinical protection from disease with repeated exposures to *Plasmodium* parasites.^15^ As children in endemic areas age and experience more parasitic exposures, they can develop naturally acquired immunity,^164^ first becoming protected from severe malaria early in childhood, and eventually from most symptomatic disease later in adolescence.^15^ Both T and B cell memory development has been measured in both mouse models and human malaria,^164^ but the exact mechanisms of each, and how *Plasmodium* parasites actively evade this development, are still incompletely understood.

### T cell–mediated memory

T cell–mediated memory to blood-stage *Plasmodium* parasites is less well characterized than B cell memory (see the following). *Plasmodium* blood stage*–*specific memory CD4+ T cells have been observed but can display a mixed phenotype with characteristics of Th1 cells, Tfh cells, and Tregs, and the protective ability of these polyfunctional cells is not yet known.^103^ Memory T cells with a Th1 phenotype have been observed in mouse models and appear to be protective upon secondary *Plasmodium* challenge.^130^^,^^165^ Th1 cells, in general, secrete IFNγ, which has been linked to the development of atypical memory B cells (aMBCs),^103^ potentially linking T cell–mediated immunity to dysfunction in a different immune compartment, although memory Th1 cells have been shown to promote B cell development upon reinfection.^130^ Memory T cells with a Tfh phenotype have also been observed, but their mechanism of protection against malaria is currently unknown.^103^ Access to memory T cells, which can reside within the tissues or secondary lymphoid organs, rather than in the peripheral blood, primarily complicates studies of their function and require multiple tissue samples over time to catch a peripheral response.^166^

### B cell–mediated memory

Humoral memory responses to *Plasmodium* have been more extensively characterized as antibodies produced by MBCs have been shown to be essential to the development of naturally acquired immunity over time. The repertoire of parasite-specific antibodies rapidly expands with repeated *Plasmodium* exposures to cover a broad array of parasite antigens,^167^^,^^168^ improving clinical protection. Although antibodies have been shown to play a large role in clinical immunity to malaria, MBCs seem to have a transmission-dependent development pattern, with inefficient development in high transmission settings.^169^ In settings with seasonal transmission, MBC populations develop slowly over time with age and exposure, with disproportionately low frequencies of MBCs observed after each transmission season that wane during the low exposure dry season.^168^^,^^170^ In low-transmission settings, MBCs are generated at expected frequencies and are long-lived.^171^ While this transmission-dependent development of MBCs is not yet well understood, previous work has suggested that dysregulated Tfh responses and/or inflammatory cytokines might contribute.^169^ In both human malaria^128^ and mouse models,^172^ circulating Tfh cells express a Th1-associated transcription factor, T-bet, after acute malaria, and this phenotype has been linked to reduced support for MBC generation. IFNγ has also been shown in mouse models to directly impair the germinal center reaction,^173^ which is key to MBC development. Further work is important to determine the mechanisms by which either or both mechanisms lead to impaired MBC generation during malaria.

### Atypical MBCs

In addition to decreased production of MBCs, persistent malaria exposure is also associated with development of MBCs with an atypical phenotype and function, termed aMBCs, although their exact role in protection from or pathogenesis of malaria is not yet well characterized.^169^ The prevalence of aMBCs in the blood is associated with the level of parasite exposure^174^ and decreases rapidly if parasite exposure is discontinued.^175^ These cells are missing the classic surface markers of MBCs, CD21 and CD27, and express less IgG on their surface but instead express the inhibitory receptor FcRL5 and the transcription factor T-bet.^169^ Interestingly, malaria-associated aMBCs show signs of hyperactivation and are resistant to further stimulation prompting some to propose that the expression of FcRL5 may be an attempt to downregulate this hyperactivation.^176^ Compared with MBCs, these cells have been shown to have reduced B cell receptor signaling capacity, reduced proliferation and are deficient in secretion of protective antibodies.^169^ The fact that aMBCs increase in frequency over time and exposure could suggest that their generation is a way for *Plasmodium* parasites to actively evade the adaptive immune system via immune dysregulation.^169^

Contrary to early studies looking at total aMBCs, recent work by Hopp et al.^177^ showed that *P. falciparum* antigen-specific aMBCs expand during acute malaria and although these cells are transcriptionally distinct at healthy baseline, they are transcriptionally similar to B cells after activation. Additionally, aMBCs were shown to express markers indicative of T cell interactions and to secrete IgG and IgM.^177^ Taken together, this new work suggests that these cells may help rather than hinder the anti-malarial immune response and warrants further study.

## *Plasmodium* dysregulation and evasion of the adaptive immune response

As an intracellular pathogen during most of its life cycle, *Plasmodium* evades some detection by the adaptive immune system simply by residing inside a host cell, particularly an RBC. Typically, intracellular pathogens are detected by CD8+ T cells, which recognize foreign antigens presented by MHC class I on the cell surface.^137^ Because mature RBCs do not express MHC class I,^85^  *Plasmodium* species that infect mature RBCs (e.g. *P. falciparum*) cannot be detected by the body’s classic anti-intracellular pathogen response. Inside the RBC, parasites are also protected from recognition by antibodies specific to antigens on the parasite surface that are not displayed on the RBC surface.^85^

In addition to an intracellular niche, *Plasmodium* parasites evade the adaptive immune response via variant surface antigen (VSA) expression and active dysregulation of the immune response to render it inefficient.^85^ These mechanisms have been most well-characterized for *P. falciparum*. Parasites decorate the surface of iRBCs with VSAs from large multigene families, and these surface antigens are the target of many anti-*Plasmodium* antibodies. However, extreme sequence variation both within and between parasite populations renders it nearly impossible to generate antibodies to all copies at once.^85^ The VSA PfEMP1 is encoded by the *var* multi-gene family, which has approximately 60 antigenically distinct copies per parasite genome and is the most well-studied VSA.^11^  *P. falciparum* also express VSAs from the RIFIN (repetitive interspersed families of polypeptide) and STEVOR (subtelomeric variant open reading frame) families, which are less well studied.^86^ While individual parasites express only 1 copy of PfEMP1 at a time, they possess the ability to switch between copies during an infection, referred to as antigenic variation, allowing for effective evasion of any antibodies generated to the previous copy (Fig. 2A).^86^ Additionally, parasites can generate new PfEMP1 sequences via nonhomologous recombination in the mosquito, leading to a near-infinite number of possible sequences that the immune system would need to recognize to provide sterile immunity to the parasites. Tissue sequestration is an additional immune evasion mechanism employed by the parasite wherein PfEMP1 and other VSAs bind to host endothelial receptors (EPCR, CD36, CSA, or intercellular adhesion molecules) to promote adherence and sequestration of iRBCs within the vasculature where they are protected from circulating immune cells and splenic clearance.^11^ Interestingly, VSAs are also capable of rosetting, in which uninfected RBCs surround an iRBC and effectively hide the PfEMP1 molecule from circulating antibodies.^86^ Taken together, parasitic expression of VSAs on the surface of iRBCs demonstrate several effective host immune evasion mechanisms.

Because DCs bridge the innate and adaptive immune systems, dysregulation of DCs can derail the entire adaptive immune response. In high transmission settings, *Plasmodium* infection has been associated with reduced DC activation, leading to impaired production of proinflammatory cytokines and T cell–activating cytokines such as IL-12, although the mechanism by which DC function is impaired is not yet known.^64^ Moreover, the precise consequences of this reduction in DC activation is not yet clear, but it may contribute to the lack of development of long-term immunity, typically associated with memory adaptive cells to this parasite. PfEMP1 can bind to CD36 or CD51 on the surface of DCs, which reduces their ability to present antigens and secrete cytokines, rather than trigger phagocytosis and antigen processing, which are key to initiating the adaptive response,^86^ potentially contributing to this dysregulation in the setting of *P. falciparum* infection (Fig. 2B). These dysregulated DCs also downregulate costimulatory molecules and secrete anti-inflammatory cytokines, further impairing their ability to initiate and/or actively suppressing an effective adaptive response.^74^^,^^178^ In addition to diminished numbers and functional dysregulation, *P. falciparum* infection has been shown to increase the susceptibility of DCs to apoptosis, which is associated with increased levels of IL-10.^71^

*Plasmodium* parasites appear to evade T cell–mediated immunity by skewing T cell polarization toward subtypes that impair the immune response.^137^ DCs with impaired activation and expression of costimulatory molecules, described previously, are less able to form stable interactions with T cells that would lead to their activation and proliferation.^178^ This ultimately can impair T cell development toward an effective anti-malarial phenotype, as has been described for Tfh cells,^179^ which are essential for the development of a B cell response.^64^ Some studies have found stable interactions between DCs and T cells but described a skewed response toward a Th1 phenotype, rather than Tfh, contributing to an impaired humoral response.^178^ Additionally, *P. berghei* has been shown to actively disrupt memory T cell development by secreting a cytokine-like molecule, *Plasmodium* macrophage migration inhibitory factor.^180^ This *Plasmodium* cytokine induces release of inflammatory cytokines from macrophages and DCs, such as TNFα and IL-12, which skew antigen-specific T cell polarization toward a short-lived effector phenotype, rather than toward a memory phenotype (Fig. 2C).^180^ Further studies of human-infecting parasites are needed to confirm whether *Plasmodium* macrophage migration inhibitory factor impacts memory T cell development in humans, as well.

In addition to impaired T cell help in the germinal center and antigenic variation, described previously, *Plasmodium* parasites may also directly dysregulate the B cell response to evade the humoral immune system. Similar to DCs, PfEMP1 can directly interact with B cells, which leads to a polyclonal, nonspecific expansion, rather than a productive, antigen-specific development.^155^ Similar to T cells, *Plasmodium* infection can also lead to polarization of B cells away from an effective, long-lasting response. *Plasmodium* infection seems to disproportionately skew the humoral response toward short-lived plasmablast generation (Fig. 2D),^136^ potentially via the highly proinflammatory environment that is characteristic of malaria.^14^ In addition to being short-lived, many of the plasmablasts induced during malaria are not specific to *Plasmodium* antigens,^14^ and these cells can act as a nutrient sink,^136^ limiting the ability of GCBs to develop and mature into specific, long-lasting effector cells. The GCBs that do manage to develop into long-lasting MBCs often develop an atypical phenotype,^174^ described in more detail previously, which may or may not lead to protection from subsequent *Plasmodium* challenges.

## Summary and conclusion

The immune response to *P. falciparum* malaria is complex and both the innate and the adaptive immune system contribute to protection from disease, as well as the development of malaria symptoms, summarized in Table 1. Additionally, *P. falciparum* has evolved numerous ways to evade the human defenses during malaria that help to shape the immune response. Here, we reviewed the major cell types involved in the human immune response to *P. falciparum* blood-stage parasites and the *P. falciparum* evasion mechanisms used to escape immune clearance. Despite decades of progress summarized here, the mechanisms of both host defenses and pathogen immune evasion are still incompletely understood. Future work is critical to characterize how *P. falciparum* shapes the human immune response, and particularly how we can exploit this information to generate stronger, long-lasting immunity to these parasites.

**Table 1:** Summary of host responses and *P. falciparum* immune evasion techniques.

Immune cell type	Host response	*P. falciparum* evasion
**Innate immune system**		
Neutrophils	Phagocytosis of merozoites or iRBCs Antibody-dependent respiratory burst NET formation	Recruitment of C1-INH, factor H and plasminogen Surface antigenic variation Development within RBCs Rosette formation to avoid phagocytosis Tissue sequestration iRBC CD47 surface expression Hemozoin formation Dysregulation of DCs (mechanism still uncharacterized)
Monocytes/macrophages	Phagocytosis (opsonic or nonopsonic) of merozoites or iRBCs Release of proinflammatory cytokines	
DCs	Bridge innate and adaptive response Phagocytosis of merozoites or iRBCs Prime T cell response in secondary lymphoid tissues	
**Adaptive immune system**		
NK cells (innate lymphoid cells)	Antibody-dependent cell-mediated cytotoxicity of iRBCs iRBC destruction via IFNγ production	Development within RBCs (no MHC class I expression) Surface antigenic variation Rosette formation to avoid antibody detection of surface antigens Impaired T cell activation via dysregulated DC response T cell polarization away from phenotypes conducive to clearing parasitemia and/or memory T cell formation Polarization of B cells away from an effective, long-lasting response
CD4+ T cells	Role depends on phenotype Activation of phagocytes via IFNγ production Reduction of immunopathology via IL-10 production B-cell support via IL-4 production	
Tfh cells	Bridge cellular and humoral response Stimulate B cell maturation and survival in germinal centers Promote B cell–mediated generation of *P. falciparum–*specific antibodies	
Tregs	Regulate inflammation via IL-10 production Antagonize control of parasitemia	
γδ T cells	Proinflammatory cytokine production Cytotoxic granule release to inhibit parasite growth in iRBCs Merozoite destruction via IFNγ production	
B cells	Generation and secretion of *P. falciparum–*specific antibodies Plasmablasts act as a nutrient sink Long-lived plasma cells continuously produce anti-*Plasmodium* antibodies	
Memory T cells	Can expand quickly to provide a faster adaptive immune response upon rechallenge with *P. falciparum* Function depends on phenotype	
Memory B cells	Can expand quickly upon rechallenge to produce anti-*Plasmodium* antibodies Can display exhausted phenotype (atypical memory B cells) with reduced B cell receptor signaling capacity	

## Data Availability

All information cited in the above text is from available published scientific literature.

## References

[1] Phillips MA et al Malaria. Nat Rev Dis Primers. 2017;3:17050. 10.1038/nrdp.2017.50 28770814

[2] World Health Organization. World Malaria Report 2022. WHO Press; 2022.

[3] Buck E , Finnigan NA. Malaria. StatPearls Publishing; 2023. 31869175

[4] Bartoloni A , Zammarchi L. Clinical aspects of uncomplicated and severe malaria. Mediterr J Hematol Infect Dis. 2012;4:e2012026. 10.4084/MJHID.2012.026 PMC3375727 22708041

[5] Milner DA Jr . Malaria pathogenesis. Cold Spring Harb Perspect Med. 2018;8:a025569. 10.1101/cshperspect.a025569 PMC5749143 28533315

[6] Lifecycle: Centers for disease control and prevention. 2020. https://www.cdc.gov/malaria/about/biology/index.html

[7] Venugopal K , Hentzschel F, Valkiunas G, Marti M. Plasmodium asexual growth and sexual development in the haematopoietic niche of the host. Nat Rev Microbiol. 2020;18:177–189. 10.1038/s41579-019-0306-2 PMC7223625 31919479

[8] Liu Z , Miao J, Cui L. Gametocytogenesis in malaria parasite: commitment, development and regulation. Future Microbiol. 2011;6:1351–1369. 10.2217/fmb.11.108 PMC5711484 22082293

[9] Bozdech Z et al The transcriptome of the intraerythrocytic developmental cycle of Plasmodium falciparum. PLoS Biol. 2003;1:e5. 10.1371/journal.pbio.0000005 PMC176545 12929205

[10] Crompton PD et al Malaria immunity in man and mosquito: insights into unsolved mysteries of a deadly infectious disease. Annu Rev Immunol 2014;32:157–187. 10.1146/annurev-immunol-032713-120220 PMC4075043 24655294

[11] Belachew EB. Immune response and evasion mechanisms of plasmodium falciparum parasites. J Immunol Res. 2018;2018:6529681. 10.1155/2018/6529681 PMC5889876 29765991

[12] Abuga KM , Jones-Warner W, Hafalla JCR. Immune responses to malaria pre-erythrocytic stages: Implications for vaccine development. Parasite Immunol. 2021;43:e12795. 10.1111/pim.12795 PMC7612353 32981095

[13] White M , Watson J. Age, exposure and immunity. Elife. 2018;7:e40150. 10.7554/eLife.40150 PMC6103766 30129437

[14] Rogers KJ , Vijay R, Butler NS. Anti-malarial humoral immunity: the long and short of it. Microbes Infect. 2021;23:104807. 10.1016/j.micinf.2021.104807 PMC8292161 33684519

[15] Langhorne J , Ndungu FM, Sponaas AM, Marsh K. Immunity to malaria: more questions than answers. Nat Immunol. 2008;9:725–732. 10.1038/ni.f.205 18563083

[16] Elvington M , Liszewski MK, Atkinson JP. Evolution of the complement system: from defense of the single cell to guardian of the intravascular space. Immunol Rev. 2016;274:9–15. 10.1111/imr.12474 PMC5108576 27782327

[17] Biryukov S , Stoute JA. Complement activation in malaria: friend or foe? Trends Mol Med. 2014;20:293–301. 10.1016/j.molmed.2014.01.001 24508275

[18] Boyle MJ et al Human antibodies fix complement to inhibit Plasmodium falciparum invasion of erythrocytes and are associated with protection against malaria. Immunity. 2015;42:580–590. 10.1016/j.immuni.2015.02.012 PMC4372259 25786180

[19] Kurtovic L et al Complement in malaria immunity and vaccines. Immunol Rev. 2020;293:38–56. 10.1111/imr.12802 PMC6972673 31556468

[20] Reiling L et al Targets of complement-fixing antibodies in protective immunity against malaria in children. Nat Commun. 2019;10:610. 10.1038/s41467-019-08528-z PMC6363798 30723225

[21] Kumaratilake LM , Ferrante A, Jaeger T, Rzepczyk CM. Effects of cytokines, complement, and antibody on the neutrophil respiratory burst and phagocytic response to Plasmodium falciparum merozoites. Infect Immun. 1992;60:3731–3738. 10.1128/iai.60.9.3731-3738.1992. PMCID: PMC257384. PMC257384 1500183

[22] Tan J , Piccoli L, Lanzavecchia A. The antibody response to plasmodium falciparum: cues for vaccine design and the discovery of receptor-based antibodies. Annu Rev Immunol. 2019;37:225–246. 10.1146/annurev-immunol-042617-053301 30566366

[23] Wiesner J et al Host cell factor CD59 restricts complement lysis of plasmodium falciparum-infected erythrocytes. Eur J Immunol. 1997;27:2708–2713. 10.1002/eji.1830271034 9368630

[24] Su XZ et al The large diverse gene family var encodes proteins involved in cytoadherence and antigenic variation of Plasmodium falciparum-infected erythrocytes. Cell. 1995;82:89–100. 10.1016/0092-8674(95)90055-1 7606788

[25] Larsen MD et al Evasion of classical complement pathway activation on plasmodium falciparum-infected erythrocytes opsonized by PfEMP1-specific IgG. Front Immunol. 2018;9:3088. 10.3389/fimmu.2018.03088 PMC6330326 30666256

[26] Akhouri RR , Goel S, Furusho H, Skoglund U, Wahlgren M. Architecture of human IgM in complex with P. falciparum erythrocyte membrane Protein 1. Cell Rep. 2016;14:723–736. 10.1016/j.celrep.2015.12.067 26776517

[27] Rasti N et al Nonimmune immunoglobulin binding and multiple adhesion characterize Plasmodium falciparum-infected erythrocytes of placental origin. Proc Natl Acad Sci U S A. 2006;103:13795–13800. 10.1073/pnas.0601519103 PMC1564255 16945914

[28] Gazzinelli RT , Kalantari P, Fitzgerald KA, Golenbock DT. Innate sensing of malaria parasites. Nat Rev Immunol. 2014;14:744–757. 10.1038/nri3742 25324127

[29] Schofield L , Hackett F. Signal transduction in host cells by a glycosylphosphatidylinositol toxin of malaria parasites. J Exp Med. 1993;177:145–153. 10.1084/jem.177.1.145 PMC2190877 8418196

[30] Nebl T , De Veer MJ, Schofield L. Stimulation of innate immune responses by malarial glycosylphosphatidylinositol via pattern recognition receptors. Parasitology. 2005;130 Suppl:S45–S62. 10.1017/S0031182005008152 16281992

[31] Parroche P et al Malaria hemozoin is immunologically inert but radically enhances innate responses by presenting malaria DNA to Toll-like receptor 9. Proc Natl Acad Sci U S A. 2007;104:1919–1924. 10.1073/pnas.0608745104 PMC1794278 17261807

[32] Kalantari P et al Dual engagement of the NLRP3 and AIM2 inflammasomes by plasmodium-derived hemozoin and DNA during malaria. Cell Rep. 2014;6:196–210. 10.1016/j.celrep.2013.12.014 PMC4105362 24388751

[33] Gallego-Marin C et al Cyclic GMP-AMP synthase is the cytosolic sensor of plasmodium falciparum genomic DNA and activates Type I IFN in Malaria. J Immunol. 2018;200:768–774. 10.4049/jimmunol.1701048 PMC5912257 29212905

[34] Pohl K , Cockburn IA. Innate immunity to malaria: The good, the bad and the unknown. Front Immunol. 2022;13:914598. 10.3389/fimmu.2022.914598 PMC9437427 36059493

[35] Coch C et al Human TLR8 senses RNA from Plasmodium falciparum-infected red blood cells which is uniquely required for the IFN-gamma response in NK Cells. Front Immunol. 2019;10:371. 10.3389/fimmu.2019.00371 PMC6445952 30972055

[36] Ostendorf T et al Immune sensing of synthetic, bacterial, and protozoan RNA by toll-like receptor 8 requires coordinated processing by RNase T2 and RNase 2. Immunity. 2020;52:591–605 e6. 10.1016/j.immuni.2020.03.009 32294405

[37] Aitken EH , Alemu A, Rogerson SJ. Neutrophils and Malaria. Front Immunol. 2018;9:3005. 10.3389/fimmu.2018.03005 PMC6306064 30619354

[38] Babatunde KA , Adenuga OF. Neutrophils in malaria: a double-edged sword role. Front Immunol. 2022;13:922377. 10.3389/fimmu.2022.922377 PMC9367684 35967409

[39] Pollenus E , Gouwy M, Van den Steen PE. Neutrophils in malaria: the good, the bad or the ugly? Parasite Immunol. 2022;44:e12912. 10.1111/pim.12912 35175636

[40] Zelter T et al Neutrophils impose strong immune pressure against PfEMP1 variants implicated in cerebral malaria. EMBO Rep. 2022;23:e53641. 10.15252/embr.202153641 PMC9171683 35417070

[41] Dasari P et al Digestive vacuoles of Plasmodium falciparum are selectively phagocytosed by and impair killing function of polymorphonuclear leukocytes. Blood. 2011;118:4946–4956. 10.1182/blood-2011-05-353920 21911835

[42] Tannous S , Ghanem E. A bite to fight: front-line innate immune defenses against malaria parasites. Pathog Glob Health. 2018;112:1–12. 10.1080/20477724.2018.1429847 PMC6056835 29376476

[43] Kharazmi A , Jepsen S, Valerius NH. Polymorphonuclear leucocytes defective in oxidative metabolism inhibit in vitro growth of Plasmodium falciparum. Evidence against an oxygen-dependent mechanism. Scand J Immunol. 1984;20:93–96. 10.1111/j.1365-3083.1984.tb00981.x 6379857

[44] Kapelski S , Klockenbring T, Fischer R, Barth S, Fendel R. Assessment of the neutrophilic antibody-dependent respiratory burst (ADRB) response to Plasmodium falciparum. J Leukoc Biol. 2014;96:1131–1142. 10.1189/jlb.4A0614-283RR PMC4226792 25118179

[45] Rodrigues DAS et al CXCR4 and MIF are required for neutrophil extracellular trap release triggered by Plasmodium-infected erythrocytes. PLoS Pathog. 2020;16:e1008230. 10.1371/journal.ppat.1008230 PMC7449500 32797076

[46] Baker VS et al Cytokine-associated neutrophil extracellular traps and antinuclear antibodies in Plasmodium falciparum infected children under six years of age. Malar J. 2008;7:41. 10.1186/1475-2875-7-41 PMC2275287 18312656

[47] Hoppenbrouwers T et al In vitro induction of NETosis: Comprehensive live imaging comparison and systematic review. PLoS One 2017;12:e0176472. 10.1371/journal.pone.0176472 PMC5423591 28486563

[48] Dunst J , Kamena F, Matuschewski K. Cytokines and Chemokines in cerebral malaria pathogenesis. Front Cell Infect Microbiol. 2017;7:324. 10.3389/fcimb.2017.00324 PMC5517394 28775960

[49] Kho S et al Circulating neutrophil extracellular traps and neutrophil activation are increased in proportion to disease severity in human malaria. J Infect Dis. 2019;219:1994–2004. 10.1093/infdis/jiy661 PMC6542661 30452670

[50] Knackstedt SL et al Neutrophil extracellular traps drive inflammatory pathogenesis in malaria. Sci Immunol. 2019;4:eaaw0336. 10.1126/sciimmunol.aaw0336 PMC6892640 31628160

[51] Mahanta A , Kar SK, Kakati S, Baruah S. Heightened inflammation in severe malaria is associated with decreased IL-10 expression levels and neutrophils. Innate Immun. 2015;21:546–552. 10.1177/1753425914561277 25466232

[52] Ioannidis LJ et al Monocyte- and Neutrophil-derived CXCL10 impairs efficient control of blood-stage malaria infection and promotes severe disease. J Immunol. 2016;196:1227–1238. 10.4049/jimmunol.1501562 26718341

[53] Zhou J et al CD14(hi)CD16+ monocytes phagocytose antibody-opsonised Plasmodium falciparum infected erythrocytes more efficiently than other monocyte subsets, and require CD16 and complement to do so. BMC Med. 2015;13:290. 10.1186/s12916-015-0391-7 PMC4493812 26149666

[54] Dobbs KR et al Monocyte dysregulation and systemic inflammation during pediatric falciparum malaria. JCI Insight. 2017;2:e95352. 10.1172/jci.insight.95352 PMC5621919 28931756

[55] Chua CLL , Ng IMJ, Yap BJM, Teo A. Factors influencing phagocytosis of malaria parasites: the story so far. Malar J. 2021;20:319. 10.1186/s12936-021-03849-1 PMC8284020 34271941

[56] Erdman LK et al CD36 and TLR interactions in inflammation and phagocytosis: implications for malaria. J Immunol. 2009;183:6452–6459. 10.4049/jimmunol.0901374 PMC2853812 19864601

[57] Tamm A , Schmidt RE. IgG binding sites on human Fc gamma receptors. Int Rev Immunol. 1997;16:57–85. 10.3109/08830189709045703 9651786

[58] Prato M , Gallo V, Giribaldi G, Arese P. Phagocytosis of haemozoin (malarial pigment) enhances metalloproteinase-9 activity in human adherent monocytes: role of IL-1beta and 15-HETE. Malar J. 2008;7:157. 10.1186/1475-2875-7-157 PMC2529304 18710562

[59] Ayi K , Patel SN, Serghides L, Smith TG, Kain KC. Nonopsonic phagocytosis of erythrocytes infected with ring-stage Plasmodium falciparum. Infect Immun. 2005;73:2559–2563. 10.1128/IAI.73.4.2559-2563.2005 PMC1087431 15784606

[60] Zhou J , Ludlow LE, Hasang W, Rogerson SJ, Jaworowski A. Opsonization of malaria-infected erythrocytes activates the inflammasome and enhances inflammatory cytokine secretion by human macrophages. Malar J. 2012;11:343. 10.1186/1475-2875-11-343 PMC3528456 23046548

[61] Waddell SJ et al Dissecting interferon-induced transcriptional programs in human peripheral blood cells. PLoS One. 2010;5:e9753. 10.1371/journal.pone.0009753 PMC2842296 20339534

[62] D’Ombrain MC et al Association of early interferon-gamma production with immunity to clinical malaria: a longitudinal study among Papua New Guinean children. Clin Infect Dis. 2008;47:1380–1387. 10.1086/592971 18947328

[63] Shrivastava SK et al Uptake of parasite-derived vesicles by astrocytes and microglial phagocytosis of infected erythrocytes may drive neuroinflammation in cerebral malaria. Glia. 2017;65:75–92. 10.1002/glia.23075 27696532

[64] Yap XZ , Lundie RJ, Beeson JG, O’Keeffe M. Dendritic cell responses and function in Malaria. Front Immunol. 2019;10:357. 10.3389/fimmu.2019.00357 PMC6409297 30886619

[65] Lundie RJ et al Blood-stage Plasmodium infection induces CD8+ T lymphocytes to parasite-expressed antigens, largely regulated by CD8alpha+ dendritic cells. Proc Natl Acad Sci U S A. 2008;105:14509–14514. 10.1073/pnas.0806727105 PMC2567226 18799734

[66] Sponaas AM et al Malaria infection changes the ability of splenic dendritic cell populations to stimulate antigen-specific T cells. J Exp Med. 2006;203:1427–1433. 10.1084/jem.20052450 PMC2118320 16754719

[67] Urban BC et al The frequency of BDCA3-positive dendritic cells is increased in the peripheral circulation of Kenyan children with severe malaria. Infect Immun. 2006;74:6700–6706. 10.1128/IAI.00861-06 PMC1698077 17000725

[68] Arama C et al Interethnic differences in antigen-presenting cell activation and TLR responses in Malian children during Plasmodium falciparum malaria. PLoS One. 2011;6:e18319. 10.1371/journal.pone.0018319 PMC3069068 21483827

[69] Guermonprez P et al Inflammatory Flt3l is essential to mobilize dendritic cells and for T cell responses during Plasmodium infection. Nat Med. 2013;19:730–738. 10.1038/nm.3197 PMC3914008 23685841

[70] Urban BC et al Peripheral blood dendritic cells in children with acute Plasmodium falciparum malaria. Blood. 2001;98:2859–2861. 10.1182/blood.v98.9.2859 11675362

[71] Pinzon-Charry A et al Apoptosis and dysfunction of blood dendritic cells in patients with falciparum and vivax malaria. J Exp Med. 2013;210:1635–1646. 10.1084/jem.20121972 PMC3727318 23835848

[72] Loughland JR et al Profoundly reduced CD1c+ myeloid dendritic cell HLA-DR and CD86 expression and increased tumor necrosis factor production in experimental human blood-stage Malaria infection. Infect Immun. 2016;84:1403–1412. 10.1128/IAI.01522-15 PMC4862702 26902728

[73] Woodberry T et al Low-level Plasmodium falciparum blood-stage infection causes dendritic cell apoptosis and dysfunction in healthy volunteers. J Infect Dis. 2012;206:333–340. 10.1093/infdis/jis366 22615323

[74] Gotz A et al Atypical activation of dendritic cells by Plasmodium falciparum. Proc Natl Acad Sci U S A. 2017;114:e10568–e10577. 10.1073/pnas.1708383114 PMC5724257 29162686

[75] Wu X , Gowda NM, Kumar S, Gowda DC. Protein-DNA complex is the exclusive malaria parasite component that activates dendritic cells and triggers innate immune responses. J Immunol. 2010;184:4338–4348. 10.4049/jimmunol.0903824 PMC2851449 20231693

[76] Hart GT et al Adaptive NK cells in people exposed to Plasmodium falciparum correlate with protection from malaria. J Exp Med. 2019;216:1280–1290. 10.1084/jem.20181681 PMC6547858 30979790

[77] Luty AJ et al Interferon-gamma responses are associated with resistance to reinfection with Plasmodium falciparum in young African children. J Infect Dis. 1999;179:980–988. 10.1086/314689 10068595

[78] McCall MB , Sauerwein RW. Interferon-γ--central mediator of protective immune responses against the pre-erythrocytic and blood stage of malaria. J Leukoc Biol. 2010;88:1131–1143. 10.1189/jlb.0310137 20610802

[79] Sekar P et al NK cell-induced damage to P.falciparum-infected erythrocytes requires ligand-specific recognition and releases parasitophorous vacuoles that are phagocytosed by monocytes in the presence of immune IgG. PLoS Pathog. 2023;19:e1011585. 10.1371/journal.ppat.1011585 PMC10659167 37939134

[80] Burrack KS , Hart GT, Hamilton SE. Contributions of natural killer cells to the immune response against Plasmodium. Malar J. 2019;18:321. 10.1186/s12936-019-2953-1 PMC6751859 31533835

[81] Moebius J et al PD-1 expression on NK Cells in Malaria-exposed individuals is associated with diminished natural cytotoxicity and enhanced antibody-dependent cellular cytotoxicity. Infect Immun. 2020;88:e00711-19. 10.1128/IAI.00711-19 PMC7035929 31907195

[82] Tukwasibwe S et al Natural killer cell antibody-dependent cellular cytotoxicity to Plasmodium falciparum is impacted by cellular phenotypes, erythrocyte polymorphisms, parasite diversity and intensity of transmission. Clin Transl Immunol. 2024;13:e70005. 10.1002/cti2.70005 PMC11528551 39493859

[83] Ty M et al Malaria-driven expansion of adaptive-like functional CD56-negative NK cells correlates with clinical immunity to malaria. Sci Transl Med. 2023;15:eadd9012. 10.1126/scitranslmed.add9012 PMC9976268 36696483

[84] Wolf A-S , Sherratt S, Riley EM. NK cells: uncertain allies against malaria. Front Immunol. 2017;8:212. 10.3389/fimmu.2017.00212 PMC5343013 28337195

[85] Gomes PS , Bhardwaj J, Rivera-Correa J, Freire-De-Lima CG, Morrot A. Immune escape strategies of Malaria parasites. Front Microbiol. 2016;7:1617. 10.3389/fmicb.2016.01617 PMC5066453 27799922

[86] Sakoguchi A , Arase H. Mechanisms for host immune evasion mediated by plasmodium falciparum-infected erythrocyte surface antigens. Front Immunol. 2022;13:901864. 10.3389/fimmu.2022.901864 PMC9240312 35784341

[87] Wright GJ , Rayner JC. Plasmodium falciparum erythrocyte invasion: combining function with immune evasion. PLoS Pathog. 2014;10:e1003943. 10.1371/journal.ppat.1003943 PMC3961354 24651270

[88] Inklaar MR , Barillas-Mury C, Jore MM. Deceiving and escaping complement - the evasive journey of the malaria parasite. Trends Parasitol. 2022;38:962–974. 10.1016/j.pt.2022.08.013 PMC9588674 36089499

[89] Kennedy AT et al Recruitment of factor H as a novel complement evasion strategy for blood-stage plasmodium falciparum infection. J Immunol. 2016;196:1239–1248. 10.4049/jimmunol.1501581 26700768

[90] Reiss T et al Acquisition of human plasminogen facilitates complement evasion by the malaria parasite Plasmodium falciparum. Eur J Immunol. 2021;51:490–493. 10.1002/eji.202048718 33022775

[91] Fernandez-Arias C et al Malaria inhibits surface expression of complement receptor 1 in monocytes/macrophages, causing decreased immune complex internalization. J Immunol. 2013;190:3363–3372. 10.4049/jimmunol.1103812 PMC3673585 23440418

[92] Deroost K , Pham TT, Opdenakker G, Van den Steen PE. The immunological balance between host and parasite in malaria. FEMS Microbiol Rev. 2016;40:208–257. 10.1093/femsre/fuv046 26657789

[93] Berendt AR , Ferguson DJ, Newbold CI. Sequestration in Plasmodium falciparum malaria: sticky cells and sticky problems. Parasitol Today. 1990;6:247–254. 10.1016/0169-4758(90)90184-6 15463355

[94] Oyong DA et al Loss of complement regulatory proteins on uninfected erythrocytes in vivax and falciparum malaria anemia. JCI Insight. 2018;3:e124854. 10.1172/jci.insight.124854 PMC6303009 30429373

[95] Lee WC et al Plasmodium-infected erythrocytes induce secretion of IGFBP7 to form type II rosettes and escape phagocytosis. Elife. 2020;9:e51546. 10.7554/eLife.51546 PMC7048393 32066522

[96] Schwarzer E et al Impairment of macrophage functions after ingestion of Plasmodium falciparum-infected erythrocytes or isolated malarial pigment. J Exp Med. 1992;176:1033–1041. 10.1084/jem.176.4.1033 PMC2119406 1402649

[97] Cunnington AJ et al Prolonged neutrophil dysfunction after Plasmodium falciparum malaria is related to hemolysis and heme oxygenase-1 induction. J Immunol. 2012;189:5336–5346. 10.4049/jimmunol.1201028 PMC3504608 23100518

[98] Bujila I et al Malaria-derived hemozoin exerts early modulatory effects on the phenotype and maturation of human dendritic cells. Cell Microbiol. 2016;18:413–423. 10.1111/cmi.12521 26348250

[99] Urban BC et al Plasmodium falciparum-infected erythrocytes modulate the maturation of dendritic cells. Nature. 1999;400:73–77. 10.1038/21900 10403251

[100] Elliott SR et al Inhibition of dendritic cell maturation by malaria is dose dependent and does not require Plasmodium falciparum erythrocyte membrane protein 1. Infect Immun. 2007;75:3621–3632. 10.1128/IAI.00095-07 PMC1932960 17470539

[101] Duffy PE , Sahu T, Akue A, Milman N, Anderson C. Pre-erythrocytic malaria vaccines: identifying the targets. Expert Rev Vaccines. 2012;11:1261–1280. 10.1586/erv.12.92 PMC3584156 23176657

[102] Kumar R , Loughland JR, Ng SS, Boyle MJ, Engwerda CR. The regulation of CD4(+) T cells during malaria. Immunol Rev. 2020;293:70–87. 10.1111/imr.12804 31674682

[103] Kurup SP , Butler NS, Harty JT. T cell-mediated immunity to malaria. Nat Rev Immunol. 2019;19:457–471. 10.1038/s41577-019-0158-z PMC6599480 30940932

[104] Franklin BS et al Malaria primes the innate immune response due to interferon-gamma induced enhancement of toll-like receptor expression and function. Proc Natl Acad Sci U S A. 2009;106:5789–5794. 10.1073/pnas.0809742106 PMC2657593 19297619

[105] Troye-Blomberg M et al Production of IL 2 and IFN-gamma by T cells from malaria patients in response to Plasmodium falciparum or erythrocyte antigens in vitro. J Immunol. 1985;135:3498–3504. 3930605

[106] Su Z , Stevenson MM. Central role of endogenous gamma interferon in protective immunity against blood-stage Plasmodium chabaudi AS infection. Infect Immun. 2000;68:4399–4406. 10.1128/IAI.68.8.4399-4406.2000. PMC98333 10899836

[107] Bastos KR et al Impaired macrophage responses may contribute to exacerbation of blood-stage Plasmodium chabaudi chabaudi malaria in interleukin-12-deficient mice. J Interferon Cytokine Res. 2002;22:1191–1199. 10.1089/10799900260475713 12581492

[108] Jaramillo M , Gowda DC, Radzioch D, Olivier M. Hemozoin increases IFN-gamma-inducible macrophage nitric oxide generation through extracellular signal-regulated kinase- and NF-kappa B-dependent pathways. J Immunol. 2003;171:4243–4253. 10.4049/jimmunol.171.8.4243 14530348

[109] Blanchette J , Jaramillo M, Olivier M. Signalling events involved in interferon-gamma-inducible macrophage nitric oxide generation. Immunology. 2003;108:513–522. 10.1046/j.1365-2567.2003.01620.x PMC1782926 12667213

[110] Weiss WR et al A plasmid encoding murine granulocyte-macrophage colony-stimulating factor increases protection conferred by a malaria DNA vaccine. J Immunol. 1998;161:2325–2332. 10.4049/jimmunol.161.5.2325 9725227

[111] Freitas do Rosario AP et al IL-27 promotes IL-10 production by effector Th1 CD4+ T cells: a critical mechanism for protection from severe immunopathology during malaria infection. J Immunol. 2012;188:1178–1190. 10.4049/jimmunol.1102755 PMC3272378 22205023

[112] Zander RA et al Type I interferons induce T regulatory 1 responses and restrict humoral immunity during experimental malaria. PLoS Pathog. 2016;12:e1005945. 10.1371/journal.ppat.1005945 PMC5061386 27732671

[113] Kirosingh AS et al Malaria-specific Type 1 regulatory T cells are more abundant in first pregnancies and associated with placental malaria. EBioMedicine. 2023;95:104772. 10.1016/j.ebiom.2023.104772 PMC10474374 37634385

[114] Boyle MJ et al The development of Plasmodium falciparum-specific IL10 CD4 T cells and protection from Malaria in children in an area of high malaria transmission. Front Immunol 2017;8:1329. 10.3389/fimmu.2017.01329 PMC5653696 29097996

[115] Walther M et al Distinct roles for FOXP3 and FOXP3 CD4 T cells in regulating cellular immunity to uncomplicated and severe Plasmodium falciparum malaria. PLoS Pathog. 2009;5:e1000364. 10.1371/journal.ppat.1000364 PMC2658808 19343213

[116] Jagannathan P et al IFNgamma/IL-10 co-producing cells dominate the CD4 response to malaria in highly exposed children. PLoS Pathog. 2014;10:e1003864. 10.1371/journal.ppat.1003864 PMC3887092 24415936

[117] von der Weid T , Kopf M, Kohler G, Langhorne J. The immune response to Plasmodium chabaudi malaria in interleukin-4-deficient mice. Eur J Immunol. 1994;24:2285–2293. 10.1002/eji.1830241004 7925557

[118] Shimoda K et al Lack of IL-4-induced Th2 response and IgE class switching in mice with disrupted Stat6 gene. Nature. 1996;380:630–633. 10.1038/380630a0 8602264

[119] Sandquist I , Kolls J. Update on regulation and effector functions of Th17 cells. F1000Res. 2018;7:205. 10.12688/f1000research.13020.1 PMC5820607 29527301

[120] Metenou S et al Filarial infection suppresses malaria-specific multifunctional Th1 and Th17 responses in malaria and filarial coinfections. J Immunol. 2011;186:4725–4733. 10.4049/jimmunol.1003778 PMC3407819 21411732

[121] Vinuesa CG , Cyster JG. How T cells earn the follicular rite of passage. Immunity. 2011;35:671–680. 10.1016/j.immuni.2011.11.001 22118524

[122] Perez-Mazliah D et al Disruption of IL-21 signaling affects T cell-B cell interactions and abrogates protective humoral immunity to malaria. PLoS Pathog 2015;11:e1004715. 10.1371/journal.ppat.1004715 PMC4370355 25763578

[123] Wikenheiser DJ , Ghosh D, Kennedy B, Stumhofer JS. The costimulatory molecule ICOS regulates host Th1 and follicular Th cell differentiation in response to plasmodium chabaudi chabaudi AS infection. J Immunol. 2016;196:778–791. 10.4049/jimmunol.1403206 PMC4705592 26667167

[124] Sebina I et al IFNAR1-signalling obstructs ICOS-mediated humoral immunity during non-lethal blood-stage plasmodium infection. PLoS Pathog. 2016;12:e1005999. 10.1371/journal.ppat.1005999 PMC5094753 27812214

[125] Victora GD , Nussenzweig MC. Germinal centers. Annu Rev Immunol. 2012;30:429–457. 10.1146/annurev-immunol-020711-075032 22224772

[126] Perez-Mazliah D et al Follicular helper T cells are essential for the elimination of plasmodium infection. EBioMedicine 2017;24:216–230. 10.1016/j.ebiom.2017.08.030 PMC5652023 28888925

[127] Figueiredo MM et al T follicular helper cells regulate the activation of B lymphocytes and antibody production during Plasmodium vivax infection. PLoS Pathog. 2017;13:e1006484. 10.1371/journal.ppat.1006484 PMC5519210 28700710

[128] Obeng-Adjei N et al Circulating Th1-cell-type Tfh cells that exhibit impaired B cell help are preferentially activated during acute malaria in children. Cell Rep. 2015;13:425–439. 10.1016/j.celrep.2015.09.004 PMC4607674 26440897

[129] Carpio VH et al T helper plasticity is orchestrated by STAT3, Bcl6, and Blimp-1 balancing pathology and protection in malaria. iScience. 2020;23:101310. 10.1016/j.isci.2020.101310 PMC7339051 32634740

[130] Zander RA et al Th1-like plasmodium-specific memory CD4(+) T cells support humoral immunity. Cell Rep. 2017;21:1839–1852. 10.1016/j.celrep.2017.10.077 PMC5693336 29141217

[131] Chan JA et al Th2-like T follicular helper cells promote functional antibody production during plasmodium falciparum infection. Cell Rep Med. 2020;1:100157. 10.1016/j.xcrm.2020.100157 PMC7762767 33377128

[132] Laidlaw BJ et al Interleukin-10 from CD4(+) follicular regulatory T cells promotes the germinal center response. Sci Immunol. 2017;2:eaan4767. 10.1126/sciimmunol.aan4767 PMC5846620 29054998

[133] Surette FA et al Extrafollicular CD4 T cell-derived IL-10 functions rapidly and transiently to support anti-Plasmodium humoral immunity. PLoS Pathog. 2021;17:e1009288. 10.1371/journal.ppat.1009288 PMC7880450 33529242

[134] Chan JA et al Age-dependent changes in circulating Tfh cells influence development of functional malaria antibodies in children. Nat Commun. 2022;13:4159. 10.1038/s41467-022-31880-6 PMC9293980 35851033

[135] Obeng-Adjei N et al Malaria-induced interferon-gamma drives the expansion of Tbethi atypical memory B cells. PLoS Pathog. 2017;13:e1006576. 10.1371/journal.ppat.1006576 PMC5633206 28953967

[136] Vijay R et al Infection-induced plasmablasts are a nutrient sink that impairs humoral immunity to malaria. Nat Immunol. 2020;21:790–801. 10.1038/s41590-020-0678-5 PMC7316608 32424361

[137] Janeway CJ , Travers P, Walport M, Shlomchik MJ. Immunobiology. 5th ed. Garland Science; 2001.

[138] Van Braeckel-Budimir N , Kurup SP, Harty JT. Regulatory issues in immunity to liver and blood-stage malaria. Curr Opin Immunol. 2016;42:91–97. 10.1016/j.coi.2016.06.008 27351448

[139] Walther M et al Upregulation of TGF-beta, FOXP3, and CD4+CD25+ regulatory T cells correlates with more rapid parasite growth in human malaria infection. Immunity. 2005;23:287–296. 10.1016/j.immuni.2005.08.006 16169501

[140] Quin JE et al Major transcriptional changes observed in the Fulani, an ethnic group less susceptible to malaria. Elife. 2017;6:e29156. 10.7554/eLife.29156 PMC5629023 28923166

[141] Sanou GS et al Haematological parameters, natural regulatory CD4 + CD25 + FOXP3+ T cells and gammadelta T cells among two sympatric ethnic groups having different susceptibility to malaria in Burkina Faso. BMC Res Notes. 2012;5:76. 10.1186/1756-0500-5-76 PMC3292809 22283984

[142] Li Q et al Dihydroartemisinin imposes positive and negative regulation on Treg and plasma cells via direct interaction and activation of c-Fos. Commun Biol. 2023;6:52. 10.1038/s42003-023-04454-5 PMC9842609 36646927

[143] Kurup SP et al Regulatory T cells impede acute and long-term immunity to blood-stage malaria through CTLA-4. Nat Med. 2017;23:1220–1225. 10.1038/nm.4395 PMC5649372 28892065

[144] Deroost K , Langhorne J. Gamma/Delta T cells and their role in protection against malaria. Front Immunol 2018;9:2973. 10.3389/fimmu.2018.02973 PMC6306408 30619330

[145] Howard J et al The antigen-presenting potential of Vγ9Vδ2 T cells during plasmodium falciparum blood-stage infection. J Infect Dis. 2017;215:1569–1579. 10.1093/infdis/jix149 28368498

[146] Seder RA , et al; VRC 312 Study Team. Protection against malaria by intravenous immunization with a nonreplicating sporozoite vaccine. Science. 2013;341:1359–1365. 10.1126/science.1241800 23929949

[147] Teirlinck AC et al Longevity and composition of cellular immune responses following experimental plasmodium falciparum malaria infection in humans. PLOS Pathog. 2011;7:e1002389. 10.1371/journal.ppat.1002389 PMC3228790 22144890

[148] Roussilhon C et al Human TcR gamma delta+ lymphocyte response on primary exposure to Plasmodium falciparum. Clin Exp Immunol. 1994;95:91–97. 10.1111/j.1365-2249.1994.tb06020.x PMC1534620 8287613

[149] Ho M et al Polyclonal expansion of peripheral gamma delta T cells in human Plasmodium falciparum malaria. Infect Immun. 1994;62:855–862. 10.1128/iai.62.3.855-862.1994. PMC186193 8112855

[150] Elloso MM , van der Heyde HC, Vande Waa JA, Manning DD, Weidanz WP. Inhibition of Plasmodium falciparum in vitro by human gamma delta T cells. J Immunol. 1994;153:1187–1194. 8027548

[151] Costa G et al Control of Plasmodium falciparum erythrocytic cycle: γδ T cells target the red blood cell–invasive merozoites. Blood 2011;118:6952–6962. 10.1182/blood-2011-08-376111 22045985

[152] Jagannathan P et al Vδ2+ T cell response to malaria correlates with protection from infection but is attenuated with repeated exposure. Sci Rep. 2017;7:11487. 10.1038/s41598-017-10624-3 PMC5597587 28904345

[153] Diallo H et al Longitudinal analysis of gamma delta T cell subsets during malaria infections in Malian adults. Malar J. 2019;18:69. 10.1186/s12936-019-2702-5 PMC6416881 30866943

[154] von Borstel A et al Repeated Plasmodium falciparum infection in humans drives the clonal expansion of an adaptive gammadelta T cell repertoire. Sci Transl Med. 2021;13:eabe7430. 10.1126/scitranslmed.abe7430 PMC9291638 34851691

[155] Silveira ELV , Dominguez MR, Soares IS. To B or Not to B: Understanding B cell responses in the development of malaria infection. Front Immunol. 2018;9:2961. 10.3389/fimmu.2018.02961 PMC6302011 30619319

[156] LeBien TW , Tedder TF. B lymphocytes: how they develop and function. Blood. 2008;112:1570–1580. 10.1182/blood-2008-02-078071 PMC2518873 18725575

[157] Lee MSJ et al Acute malaria suppresses the B lymphocytic niche in the bone marrow through the alteration of CXCL12-abundant reticular cells. Int Immunol. 2024;36:339–352. 10.1093/intimm/dxae012 PMC11161414 38430523

[158] Bredius RG et al Role of neutrophil Fc gamma RIIa (CD32) and Fc gamma RIIIb (CD16) polymorphic forms in phagocytosis of human IgG1- and IgG3-opsonized bacteria and erythrocytes. Immunology. 1994;83:624–630. PMC1415059 7875742

[159] Sakamoto H et al Antibodies against a Plasmodium falciparum antigen PfMSPDBL1 inhibit merozoite invasion into human erythrocytes. Vaccine. 2012;30:1972–1980. 10.1016/j.vaccine.2012.01.010 22248820

[160] Osier FH et al Opsonic phagocytosis of Plasmodium falciparum merozoites: mechanism in human immunity and a correlate of protection against malaria. BMC Med. 2014;12:108. 10.1186/1741-7015-12-108 PMC4098671 24980799

[161] Osier FH et al Breadth and magnitude of antibody responses to multiple Plasmodium falciparum merozoite antigens are associated with protection from clinical malaria. Infect Immun. 2008;76:2240–2248. 10.1128/IAI.01585-07 PMC2346713 18316390

[162] Polley SD et al Human antibodies to recombinant protein constructs of Plasmodium falciparum Apical Membrane Antigen 1 (AMA1) and their associations with protection from malaria. Vaccine. 2004;23:718–728. 10.1016/j.vaccine.2004.05.031 15542195

[163] Triglia T et al Apical membrane antigen 1 plays a central role in erythrocyte invasion by Plasmodium species. Mol Microbiol. 2000;38:706–718. 10.1046/j.1365-2958.2000.02175.x 11115107

[164] Struik SS , Riley EM. Does malaria suffer from lack of memory? Immunol Rev. 2004;201:268–290. 10.1111/j.0105-2896.2004.00181.x 15361247

[165] Opata MM et al Protection by and maintenance of CD4 effector memory and effector T cell subsets in persistent malaria infection. PLoS Pathog. 2018;14:e1006960. 10.1371/journal.ppat.1006960 PMC5908200 29630679

[166] Achtman AH , Bull PC, Stephens R, Langhorne J. Longevity of the immune response and memory to blood-stage malaria infection. In: Langhorne J, ed. Immunology and immunopathogenesis of malaria. Springer; 2005. p. 71–102. 10.1007/3-540-29967-x_3 16265903

[167] Finney OC et al Predicting antidisease immunity using proteome arrays and sera from children naturally exposed to malaria. Mol Cell Proteomics. 2014;13:2646–2660. 10.1074/mcp.M113.036632 PMC4188993 25023128

[168] Wendel BS et al Accurate immune repertoire sequencing reveals malaria infection driven antibody lineage diversification in young children. Nat Commun. 2017;8:531. 10.1038/s41467-017-00645-x PMC5599618 28912592

[169] Ly A , Hansen DS. Development of B cell memory in malaria. Front Immunol. 2019;10:559. 10.3389/fimmu.2019.00559 PMC6454213 31001244

[170] Weiss GE et al The Plasmodium falciparum-specific human memory B cell compartment expands gradually with repeated malaria infections. PLoS Pathog 2010;6:e1000912. 10.1371/journal.ppat.1000912 PMC2873912 20502681

[171] Nogaro SI et al The breadth, but not the magnitude, of circulating memory B cell responses to P. falciparum increases with age/exposure in an area of low transmission. PLoS One 2011;6:e25582. 10.1371/journal.pone.0025582 PMC3186790 21991321

[172] Ryg-Cornejo V et al Severe malaria infections impair germinal center responses by inhibiting T follicular helper cell differentiation. Cell Rep. 2016;14:68–81. 10.1016/j.celrep.2015.12.006 26725120

[173] Ryg-Cornejo V , Ly A, Hansen DS. Immunological processes underlying the slow acquisition of humoral immunity to malaria. Parasitology. 2016;143:199–207. 10.1017/S0031182015001705 26743747

[174] Weiss GE et al Atypical memory B cells are greatly expanded in individuals living in a malaria-endemic area. J Immunol. 2009;183:2176–2182. 10.4049/jimmunol.0901297 PMC2713793 19592645

[175] Ayieko C et al Changes in B cell populations and merozoite surface Protein-1-specific memory B cell responses after prolonged absence of detectable P. falciparum infection. PLoS One 2013;8:e67230. 10.1371/journal.pone.0067230 PMC3695086 23826242

[176] Braddom AE , Batugedara G, Bol S, Bunnik EM. Potential functions of atypical memory B cells in Plasmodium-exposed individuals. Int J Parasitol. 2020;50:1033–1042. 10.1016/j.ijpara.2020.08.003. PMC7666103 32987039

[177] Hopp CS et al Atypical B cells up-regulate costimulatory molecules during malaria and secrete antibodies with T follicular helper cell support. Sci Immunol. 2022;7:eabn1250. 10.1126/sciimmunol.abn1250 PMC11132112 35559666

[178] Osii RS , Otto TD, Garside P, Ndungu FM, Brewer JM. The impact of malaria parasites on dendritic cell-T cell interaction. Front Immunol. 2020;11:1597. 10.3389/fimmu.2020.01597 PMC7393936 32793231

[179] Fontana MF , Ollmann Saphire E, Pepper M. Plasmodium infection disrupts the T follicular helper cell response to heterologous immunization. Elife. 2023;12:e83330. 10.7554/eLife.83330 PMC9886276 36715223

[180] Sun T et al A Plasmodium-encoded cytokine suppresses T-cell immunity during malaria. Proc Natl Acad Sci U S A. 2012;109:e2117–e2126. 10.1073/pnas.1206573109 PMC3411961 22778413

[181] Asawa R. Innate and adaptive immune response in P. falciparum. 2025. https://BioRender.com/w47l859

[182] Asawa R. Blood-stage immune evasion mechanisms in P. falciparum. 2025. https://BioRender.com/u51r092

